# Systematic domain-based aggregation of protein structures highlights DNA-, RNA- and other ligand-binding positions

**DOI:** 10.1093/nar/gky1224

**Published:** 2018-12-07

**Authors:** Shilpa Nadimpalli Kobren, Mona Singh

**Affiliations:** 1 Department of Biomedical Informatics, Harvard Medical School, 10 Shattuck Street, Boston, MA 02115, USA; 2 Department of Computer Science, Princeton University, 35 Olden Street, Princeton, NJ 08544, USA; 3 Lewis-Sigler Institute for Integrative Genomics, Princeton University, Carl Icahn Laboratory, Princeton, NJ 08544, USA

## Abstract

Domains are fundamental subunits of proteins, and while they play major roles in facilitating protein–DNA, protein–RNA and other protein–ligand interactions, a systematic assessment of their various interaction modes is still lacking. A comprehensive resource identifying positions within domains that tend to interact with nucleic acids, small molecules and other ligands would expand our knowledge of domain functionality as well as aid in detecting ligand-binding sites within structurally uncharacterized proteins. Here, we introduce an approach to identify per-domain-position interaction ‘frequencies’ by aggregating protein co-complex structures by domain and ascertaining how often residues mapping to each domain position interact with ligands. We perform this domain-based analysis on ∼91000 co-complex structures, and infer positions involved in binding DNA, RNA, peptides, ions or small molecules across 4128 domains, which we refer to collectively as the InteracDome. Cross-validation testing reveals that ligand-binding positions for 2152 domains are highly consistent and can be used to identify residues facilitating interactions in ∼63–69% of human genes. Our resource of domain-inferred ligand-binding sites should be a great aid in understanding disease etiology: whereas these sites are enriched in Mendelian-associated and cancer somatic mutations, they are depleted in polymorphisms observed across healthy populations. The InteracDome is available at http://interacdome.princeton.edu.

## INTRODUCTION

The rate at which new genomes are sequenced has long since outpaced our ability to experimentally characterize the biological functions of the encoded genes and their protein products. Leveraging the fact that similar protein sequences or subsequences tend to share similar functions, computational approaches have been developed to mitigate this sequence-to-function discrepancy by rapidly detecting and modeling the sequence similarity between proteins (1). Such homology-driven analyses of large-scale protein sequence databases have revealed many thousands of recurrent, probabilistically modelable protein subsequences called ‘domains’ (2–4). These sequence-derived domains correspond to evolutionarily and functionally related substructures of proteins and are found in various modular combinations within proteins from species across the tree of life (5).

Individual protein domains are associated with specific functionalities, among the most important of which are mediating the interactions proteins make with nucleic acids, other proteins and various other molecules in the cell. Indeed, protein–DNA and protein–RNA interactions have been found to occur via domain interfaces so frequently that factors associated with transcriptional and post-transcriptional activity are regularly classified according to their incorporation of particular nucleic acid-binding domains (6,7). Moreover, a significant proportion of protein–protein interactions in signaling pathways are mediated by modular binding domains (8).

Although simply knowing which domains mediate various ligand interactions has already accelerated our ability to annotate protein functions (9), pinpointing the ligand-contacting positions *within* these domains would enable a more precise analysis of the many thousands of sequenced proteins across species that contain domain instances but lack further biological characterizations. Indeed, more comprehensive knowledge of protein interaction interfaces will have considerable implications for investigating the evolution and natural variation of interaction network connectivities (10), for determining the mechanistic impact of coding variants (11), for prioritizing germline and somatic perturbations to uncover disease etiology (12,13), and for designing targeted therapeutic drugs (14).

Identifying positions within domains that interact with ligands from sequence alone is nontrivial, as a minority of positions within a domain may be involved with ligand binding, and these positions may not be proximal with respect to the linear protein sequence (e.g. of 264 positions in the tyrosine kinase domain, only 16 non-contiguous positions contact adenosine triphosphate (ATP)) (15). Further, while some ligand-binding positions are largely invariant across domain instances—for example, the zinc-contacting positions in the Cys_2_-His_2_ zinc finger (C2H2-ZF) domain are required for proper domain folding and thus are highly conserved—other binding positions are not: amino acids within DNA-contacting positions in these same C2H2-ZF domains, for example, vary dramatically across domain instances to confer diverse binding specificities, and thus cannot be identified by conservation-based analyses (16).

On the other hand, analyses of three-dimensional structures of proteins co-complexed with ligands are highly accurate in identifying positions comprising interaction interfaces. Previously, co-complex structures of a single or a few manually-selected domain instances have been used as models to distinguish domain positions involved in ligand binding from those that are not (17,18). However, binary classifications of domain binding positions determined from single structures are not always generalizable; indeed, analyses of structurally distinct instances of some domain families have revealed that the positions involved in binding peptides or other domains can vary (19). As such, although various databases have associated domain families with corresponding structures and bound ligands, particularly in the context of domain–domain interactions, they have largely avoided attempts to systematically determine, across multiple ligand types, the *positions* within these domains that mediate interactions (19–23).

Here we introduce a robust, large-scale structural aggregation approach to systematically identify positions within domains that are likely to interact with ligands. Our main contributions are as follows. First, we analyze over 91000 protein–ligand co-complex structures in the context of domains and develop a proximity-based scoring function that determines real-valued ligand-binding frequencies across individual positions in 4128 domains; we compute per-position binding frequencies separately for DNA, RNA, peptide, ion, metabolite and other small molecule ligands. Second, we show via cross-validation testing that the resultant per-domain-position binding frequencies can accurately reveal positions that bind ligands in held-out structures. Third, we utilize these **Inter**action **Dom**ains, which we refer to collectively as the **InteracDome**, to infer interaction sites across ∼63% of human genes with high confidence, and up to ∼69% of human genes more broadly; this represents the most comprehensive resource of this type to date. Fourth, we uncover that these domain-inferred interaction sites across human proteins exhibit significant functional constraints: they are depleted for natural variants across healthy human populations, while they are enriched for Mendelian disease-associated and cancer somatic mutations. Finally, we conclude with a discussion of how our InteracDome resource can be leveraged to provide valuable, medically-relevant insights by detecting and interpreting the mechanistic effects of disease-associated coding mutations.

## MATERIALS AND METHODS

### Overview

**Figure 1.**
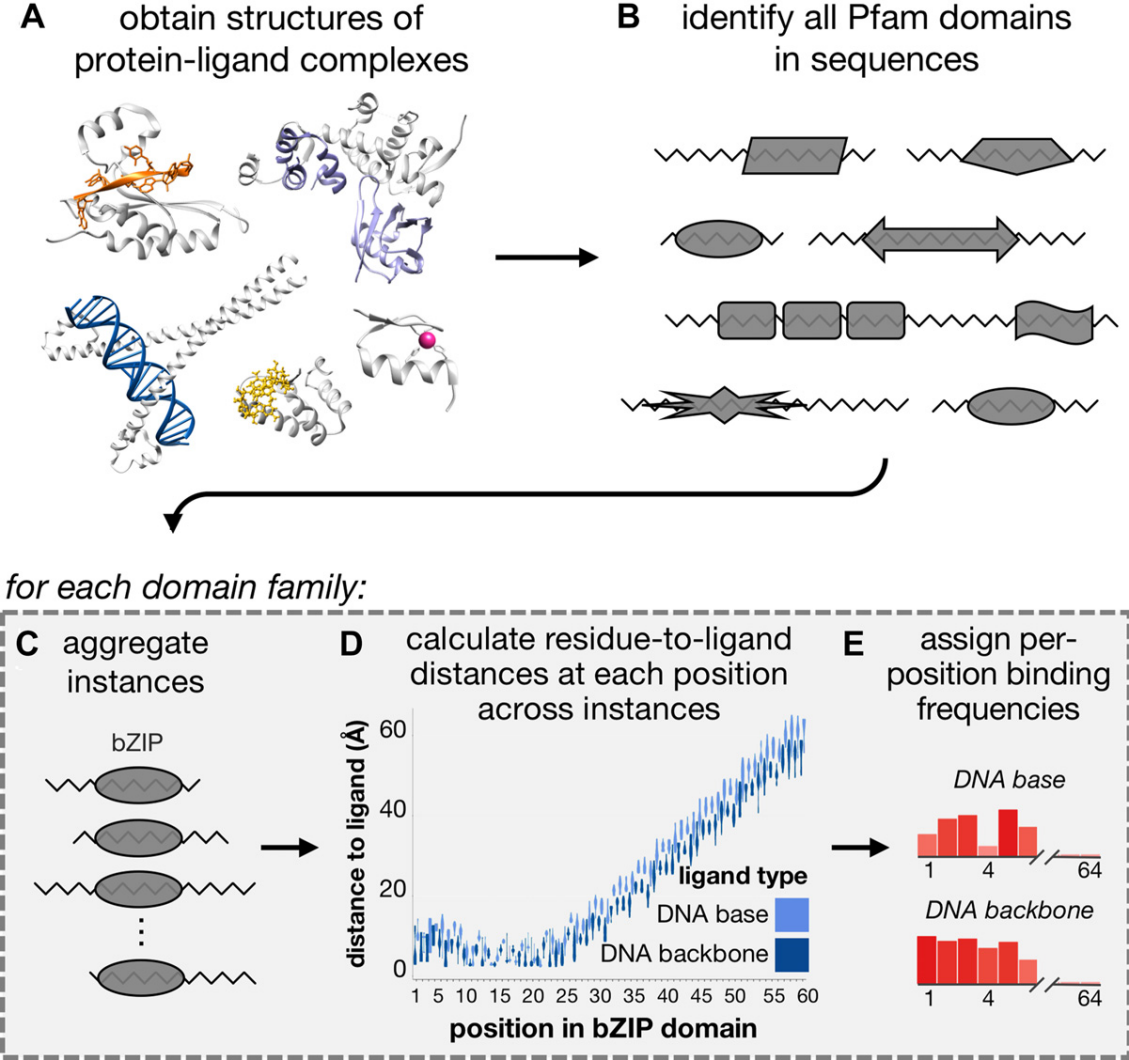
**Workflow for computing per-position binding frequencies for domains**. (**A**) Structures of protein–ligand binding complexes are obtained from BioLiP (24); pictured here are proteins in complex with DNA (blue, PDB ID: 4auw), RNA (orange, PDB ID: 5els), peptides (purple, PDB ID: 5ibk), a zinc ion (pink, PDB ID: 1aay) and the small molecule GMP (yellow, PDB ID: 5tzd). Protein chains are colored gray. (**B**) Instances of Pfam domain families are found across BioLiP structures. For each Pfam domain family found in (B), we (**C**) aggregate all instances by ligand-binding type, (**D**) calculate distributions of minimum distances from residues to ligands and (**E**) calculate a real-valued binding frequency for each domain position for each ligand type.

In this section, we describe our framework for systematically evaluating how different positions within domains are involved in mediating various ligand interactions. Briefly, we first obtain from BioLiP (24) a comprehensive collection of structures of proteins co-complexed with various ligands (Figure 1A). Each of these structures contains the three-dimensional locations of all atoms within a protein chain and all atoms within a ligand; the protein chains are also represented linearly as sequences of amino acids. We then use probabilistic sequence matching to find instances of protein domains within these protein sequences (Figure 1B). For those domain families with instances across multiple protein sequences, we aggregate their instances into a multiple sequence alignment such that each column of the alignment corresponds to a ‘core’ domain position (i.e. a match state in the corresponding HMM profile (2)), and each row corresponds to a different domain instance (Figure 1C). Next, we analyze the structure corresponding to each domain instance. For each amino acid residue in that instance corresponding to a core domain position, we calculate the minimum Euclidean distance between any of the atoms in that residue’s side chain to any atoms in a ligand. This process leaves us with a distribution of minimum residue-to-ligand distances observed across structural instances for each domain position (Figure 1D). Finally, we distill each of these per-domain-position distance distributions into a single ‘binding frequency’ value that reflects that domain position’s tendency to bind a particular ligand (Figure 1E). We describe each of these steps in more detail next.

### Aggregating structural co-complexes to identify interaction domains

We downloaded 91063 X-ray crystal and nuclear magnetic resonance (NMR) structures on 12 September 2018 from BioLiP (24), a curated database of biologically relevant protein–ligand complexes; 7095 structures contain nucleic acid molecules, 9373 contain peptides, and 82355 contain additional ligands (Figure 1A). To identify domains that mediate protein interactions, we query all BioLiP protein sequences for instances of 16712 profile Hidden Markov models from the Pfam-A database (v31.0) using HMMER (v2.3.2 and v3.1b2) (2,25). We restrict to domain instances that pass Pfam’s default gathering thresholds, have residues at the first and last domain positions, have the most likely residue at domain positions with information content ≥4 (corresponding to a distribution where the most frequent amino acid appears with ∼95% probability), and contain at least one residue annotated by BioLiP to be involved in ligand binding (Figure 1B).

### Differentiating and grouping ligand types

We classify BioLiP ligands into biologically relevant groups. Protein residues responsible for determining DNA- and RNA-binding specificity often contact nucleic acid bases, whereas residues that primarily contact the backbones of nucleic acid molecules may be more important for the stability and affinity of the binding complex (17,26). As BioLiP groups all DNA and RNA ligand molecules together, we reanalyze the original co-complex structures to characterize ligand atoms based on the presence (RNA) or absence (DNA) of the 2’-hydroxyl in the ribose sugar; these ligands respectively occur in 2718 and 4377 co-complex structures. We further group these ligand atoms into RNA base, RNA backbone, DNA base or DNA backbone.

The 123 ligands from 38234 co-complex structures with ‘ion’ in their full names are assigned to the ion group; the remaining 23829 ligands from 64918 co-complex structures are grouped as small molecules. BioLiP already excludes molecular artifacts from crystallization buffers, yet does not explicitly differentiate cognate (i.e. naturally occurring *in vivo*) from non-cognate ligands. To highlight domain positions whose residues comprise metabolically relevant and/or potentially druggable binding pockets, we further categorize small molecules as follows. Any small molecule ligand with a Tanimoto coefficient ≥ 0.9 (Open Babel Package, v2.4.1) (27,28) between its SMILES string (wwPDB’s Chemical Component Dictionary, v3.30) and the SMILES strings of one of 102 639 endogenous human metabolites (Human Metabolome Database, v4.0) (29) and/or 9296 drugs (DrugBank, v5.1) (30) is respectively classified as metabolite and/or druglike; these ligand types respectively occur in 35262 and 42947 co-complex structures. We note that previously a Tanimoto coefficient cutoff of 0.9 was shown to efficiently reduce molecular database search spaces while still enabling accurate molecule matching (31); further, we find that cutoffs as low as 0.8 to classify small molecules as metabolite and/or druglike do not substantially alter the numbers of domains found to be in complex with these ligand types.

### Computing proximity-based positional binding frequencies

We define the *representable* set of domain–ligand interactions as those with at least one corresponding domain–ligand co-complex structure in BioLiP. We require that these structures give coordinates for residue side-chain atoms—as opposed to just backbone carbon atoms—but do not otherwise consider structural resolution. For each domain in the representable set, we assess the ligand-binding frequencies of individual domain positions by aggregating protein–ligand atom proximity information across their corresponding co-complex structures (Figure 1C–E). For structures that have multiple chains or chain orientations with identical sequences, we select the single chain with the smallest Euclidean distance to the ligand of interest. For each instance of the domain in a BioLiP structure that contains at least one residue in contact with a particular ligand type, for each of its residues corresponding to a Pfam domain match state, we compute the minimum Euclidean distance between heavy (i.e. non-hydrogen) side-chain atoms to any heavy ligand atom. We aggregate these distances by domain position across all BioLiP instances for the same domain–ligand type pair, resulting in a per-domain-position distribution of minimum distances to the ligand (Figure 1C–D). We next determine what fraction of these distances are within 3.6Å of the ligand (Figure 1E); however, to proportionally minimize the contribution of structural instances with highly redundant sequences, we apply the Henikoff and Henikoff sequence weighting scheme (32) to the domain instances. Thus, intuitively, our per-domain-position binding frequency computes the (weighted) fraction of times a residue in that position is within 3.6Å of the ligand type considered. Note that the distance of 3.6Å will capture both hydrogen bonds (2.6–3.3Å) and van der Waals contacts (2.8–4.1Å), but will eliminate water-mediated interactions (as in (33)). We find that residue-to-ligand proximity cutoffs between 2.5 and 5.0Å do not substantially alter which positions have the top 10–15% of ligand-binding frequency values per domain.

### Cross-validation testing of domain-to-ligand distance consistencies and positional binding frequencies

We refer to each domain–ligand type pair with at least three distinct (i.e. non-redundant) sequences across separate PDB entries as belonging to the *representable-NR* set. For each domain–ligand pair in the representable-NR set, we evaluate both the consistency of its structural interface as well as the accuracy of our aggregation approach in identifying ligand-binding positions in cross-validation.

First, to evaluate consistencies of domain–ligand structural interfaces, for each domain–ligand pair in the representable-NR set, we first randomly split its structural instances into two folds. We next compute, across all instances within each fold, the average minimum residue-to-ligand distance at each domain position. Finally, we compute the Pearson’s correlation coefficient (PCC) between the two resulting domain-length vectors. We report the average PCC achieved across ten repetitions of this process as the consistency of the domain–ligand structural interface.

Second, to test the power of our approach in identifying binding positions across previously unseen domain instances, for each domain–ligand type pair in the representable-NR set, we randomly divide its structural instances into up to 10 folds. For each domain instance *i* in each hold-out fold in turn, we examine the structure to assign a binary vector where 1 and 0 respectively indicate domain positions whose residues are or are not in contact with the ligand (i.e. as annotated by BioLiP). Binding frequencies are then calculated as before from instances in the remaining folds. For each position in each instance in the hold-out fold, we use the corresponding positional binding frequency, computed from the other folds, as its ‘score.’ We then rank in descending order all positions within the hold-out fold by score, with higher ranking positions corresponding to the more confident predictions of binding. As we iteratively decrease the score threshold used to predict whether a position is binding, we compute precision and recall with respect to the known binding (true positive) and non-binding (true negative) positions inferred from the actual structures in the held-out set. This allows us to compute a precision-recall curve (PRC) for each domain–ligand interaction. We refer to the set of domain–ligand interactions that achieved a cross-validated precision of at least 0.5 at some threshold as the *confident* set. We also compute the area under under the PRC (AUPRC), and compare it to an average baseline AUPRC corresponding to the fraction of binding positions in the held-out set.

We note that instances of the same domain family have by definition clearly identifiable sequence similarity and thus can have highly similar amino acid sequences. Nevertheless, as an alternate cross-validation test, for each domain–ligand type pair in the representable-NR set, we also try to divide all its instances within BioLiP into groups such that the amino acid sequence identity between instances in different groups is <90%. We repeat the steps above to determine domain–ligand structural consistencies and cross-validated precisions and recalls by dividing these groups of BioLiP instances with sequence identity ≥90%—rather than individual instances—into folds as before; these results are reported in the ‘Supplementary Data’ section.

### Human protein, natural variation and disease mutation datasets

Protein sequences, corresponding cDNA sequences and corresponding genomic coordinates for 104 295 known and predicted human protein isoforms encoded by 23043 genes were downloaded from Ensembl (build GRCh38.p10). We consider the subset of 89024 protein isoforms from 22712 human genes where the genomic DNA sequence matched the cDNA sequence, the cDNA sequence translated to the protein sequence with ≤5% sequence mismatch, and the protein transcript was not annotated with ‘decay’ nor ‘pseudogene.’ We functionally classify single nucleotide variants (SNVs) with respect to the longest protein isoform for each gene by mapping SNVs onto Ensembl cDNA sequences and translating to proteins.

Naturally occurring exonic SNVs from 123 136 healthy humans were downloaded from the Genome Aggregation database (gnomAD, v2.0.2) (34). We restrict to the set of 194 868 common missense SNVs that are found with frequency ≥ 0.001 across any gnomAD subpopulation. We also obtained 28242 missense germline disease mutations affecting 24823 sites across the canonical protein isoforms of 2590 human genes from UniProtKB’s Humsavar database (v2017_04) (35) and augmented this set with an additional 1912 validated missense germline disease mutations occurring an additional 159 human genes from OMIM (v2011_02) (http://www.bioinf.org.uk/omim/).

We also downloaded all open-access TCGA somatic SNV data and RNA-seq expression data from NCI’s Genomic Data Commons on 15 July 2017 (36,37). We exclude all SNVs occurring after a frameshift or nonsense mutation in the corresponding tumor sample and all SNVs from genes that were expressed at <0.1 TPM (in the corresponding tumor sample or on average across other tumor samples of the same tissue type when expression data was missing). These steps resulted in a filtered set of 1 171 890 missense somatic SNVs across 18627 genes using data from 10037 tumors across 33 cancer types. Finally, 1209 known cancer driver missense SNVs were downloaded from the Database of Curated Mutations (DoCM, v3.2) (38).

### Inferring putative ligand-binding positions in human proteins

We query the longest protein isoform of each human gene and infer ligand-binding positions in these proteins in three ways. First, we extract human proteins from BioLiP, and obtain the residues identified in this database to interact with ligands. Next, we transfer structural binding information from BioLiP to human proteins with high sequence similarity, as described previously (13). Finally, for each domain where we have estimated per-position binding frequencies, we find matches to this domain in human sequences using HMMER as described above and transfer the ligand-binding frequencies to any protein residue that corresponds to a core domain position. In practice for this last step, only domain–ligand pairs from the confident set are used. For each of these domain–ligand pairs, the threshold to define ligand-binding positions is chosen as the value that resulted in cross-validated precision ≥0.5, as described above.

### Determining significance of overlap with inferred ligand-binding sites

Given a set of sites of interest in human proteins (e.g. sites harboring common missense SNVs across populations) and a set of putative ligand-binding sites in human proteins, we determine whether the overlap between these two sets is significantly larger or smaller than what is expected by chance alone using the Poisson binomial distribution. Here, the *N* sites of interest across proteins are modeled as *N* independent Bernoulli trials, where the *p*_1_, ..., *p*_*N*_ probabilities of ‘success’ (i.e. overlap with the putative binding sites) for each trial are non-uniform. Specifically, each success probability *p*_*i*_ is equal to the proportion of putative binding sites in the protein where the site of interest *i* occurs; this way, sites of interest that occur in proteins with a large proportion of putative ligand-binding sites will not bias global trends. We determine if *K*—the number of sites of interest observed to overlap with the putative binding sites—is significantly greater than or less than we would expect by chance by respectively computing Pr(*X* ≥ *K*) and Pr(*X* ≤ *K*) using the Poisson binomial implemented in R’s poibin package (39). *P*-values computed as 0 are reported as 1e-15, the lowest non-zero *P*-value we achieved using poibin.

## RESULTS

Our fully automated procedure to build the InteracDome resource identifies 4128 domain families that are representable (i.e. have ligand interactions across one or more instances in structural co-complexes). Of these, 2375 domain families have at least three non-redundant instances in complex with the same ligand type across distinct PDB structures; these domain–ligand interactions comprise the representable-NR set. Within the representable set of domain interactions, 564 domain families are co-complexed with DNA, 490 are co-complexed with RNA, 830 are co-complexed with other peptides, 2557 are co-complexed with ions and 2847 are co-complexed with one or more small molecules. In the representable-NR set, 257, 244, 299, 1204 and 1634 domain families are respectively co-complexed with DNA, RNA, peptides, ions and/or small molecules. Note that the same domain family can be found to interact with multiple ligand types (e.g. the RRM domain interacts with both DNA and RNA across instances).

### Case studies: InteracDome includes well-known interaction domains and recapitulates known ligand-binding domain positions

We begin by ascertaining how well the interaction domains profiled in the InteracDome cover known ligand-binding domains. Toward this end, we compiled a list of 54 DNA-binding domain families from the Thornton Lab review (40), 12 RNA-binding domain families from the review by (41) and 78 human peptide-binding domain families listed on the Pawson Lab site (http://pawsonlab.mshri.on.ca). We find that our representable set of domain–ligand interactions includes all the DNA-binding domain families, all the RNA-binding domain families and ∼85% of postulated protein-binding domain families, many of which have been particularly difficult to structurally characterize due to the low affinity and transience of protein–protein interactions in signaling pathways (42). Of these known ligand-binding domains, 42 (78%) DNA-binding domains, 9 (75%) RNA-binding domains and 47 (60%) peptide-binding domains are found in the representable-NR set; these numbers are 42 (78%), 8 (67%) and 43 (55%), respectively, for known ligand-binding domains found in the confident set.

Next, we turn our attention to how well our per-domain-position binding frequencies identify manually curated ligand-binding domain positions. In particular, domain positions involved in ligand binding have previously been established for a few well-studied domains using one or a few structural co-complexes. Intuitively, our method automates this approach at a much larger scale; thus, we expect that domain positions assigned high binding frequencies by our method will largely be in agreement with previous knowledge of domain binding. We highlight below a few well-studied nucleic acid-, peptide- and metabolite-binding domains to show that indeed, when we compare InteracDome binding frequencies with literature-curated knowledge of domain–ligand binding, domain positions with high binding frequency values recapitulate known interaction positions.

**Figure 2.**
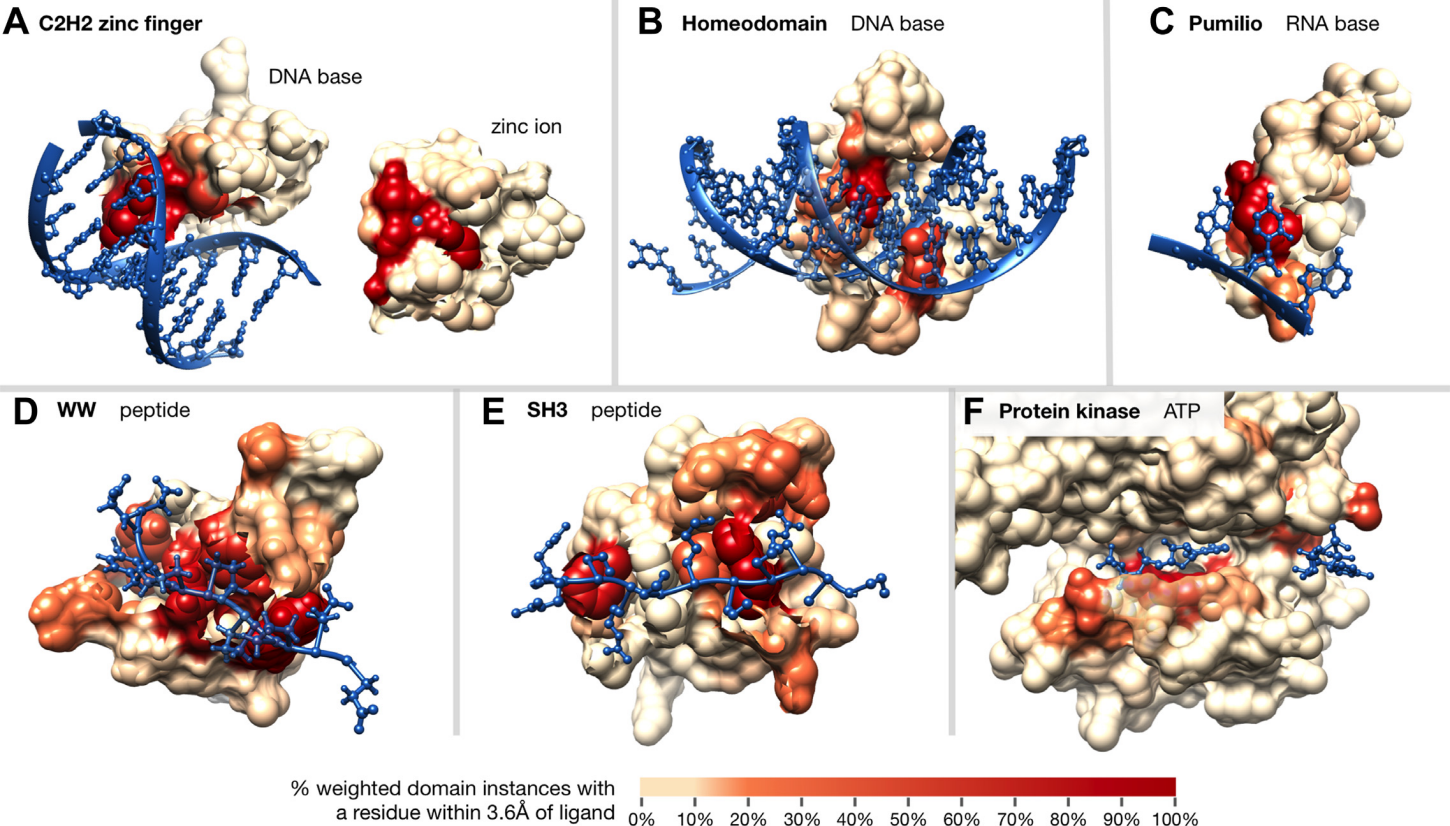
**Examples of domains scored according to ligand-binding frequency**. Residues within interaction domain structures are colored according to their ligand-binding frequencies; domains pictured include: (**A**) C2H2-ZF domain (PF00096, PDB ID: 1aay, second domain of chain A), the zinc ion is shown on top of the domain for visibility, (**B**) Homeodomain (PF00046, PDB ID: 1ig7), (**C**) Pumilio domain (PF00806, PDB ID: 1m8w, third domain of chain B), (**D**) WW domain (PF00397, PDB ID: 2n1o), (**E**) SH3 domain (PF00018, PDB ID: 2bz8) and (**F**) protein kinase domain (PF00069, PDB ID: 1csn, subdomains I–V, both ATP molecules from two units shown).

We first consider three nucleic acid binding domains. The C2H2-ZF domain is known to specify its DNA targets via four DNA-base contacting positions (-1, 2, 3 and 6 in the α-helix contacting DNA) (33); these four positions have the highest DNA base-binding frequencies for this domain in the InteracDome. Additionally, there are two highly conserved cysteines and histidines that coordinate the zinc ion—required for proper domain folding—and these four positions have our highest ion-binding frequencies (Figure 2A and Supplementary Figure S1a). In the DNA-binding homeodomain, our highest DNA base-binding frequencies correspond to positions 45–46, 49–50 and 53–54 in the DNA recognition helix, followed by positions 1–4 in the N-terminal arm (Figure 2B and Supplementary Figure S1b); these are known specificity-determining positions in the domain (18,43). In the RNA-binding pumilio domain, the highest RNA base-binding frequencies are found in positions 14, 16, 17 and 20 of the repeating α-helix section (Figure 2C and Supplementary Figure S1c). Indeed, positions 16, 17 and 20 confer RNA-binding specificity, and position 14 contacts RNA backbone ribose rings, likely affecting binding (26).

We next examine InteracDome binding frequencies for two peptide-binding domains. In the WW domain, our highest peptide-binding frequencies are found at positions 17, 19, 21, 24, 26 and 28—all corresponding to known binding residues. The next highest peptide-binding frequencies identify positions 8, 10 and 11—all known to confer binding specificity differences between type I and IV domains (Figure 2D and Supplementary Figure S1d) (44). Sites with high binding frequencies for the SH3 domain are also relevant for peptide binding. In particular, positions with the highest binding frequency values are 4, 6, 32 and 47—the four most conserved peptide-binding sites—and positions 9–13, 27–30, 34 and 45—all known to be important for distinct peptide-binding specificities (Figure 2E and Supplementary Figure S1e) (45).

Finally, our approach also recapitulates ATP-binding positions in the kinase domain. Our high small molecule binding frequencies include several residues from subdomains I–VII that are responsible for interacting with and anchoring ATP’s adenine ring (i.e. positions 7, 15, 28, 77–80, 84), α, β and γ phosphates (i.e. positions 11–13, 30, 128, 141), and ribose hydroxyl group (i.e. position 127) (15). We also highly rank an additional six sites within three amino acids of a known binding position (Figure 2F and Supplementary Figure S1f).

### Domain-to-ligand proximities are consistent across instances

**Figure 3.**
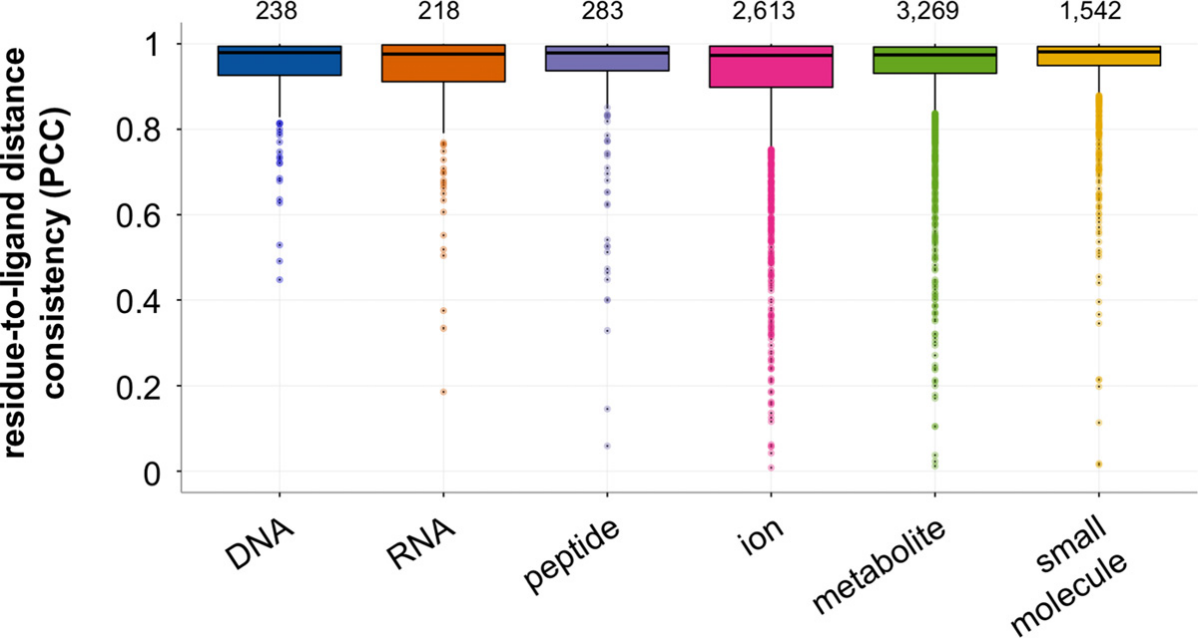
**Domain-to-ligand distance consistencies**. Structural instances across BioLiP for each domain–ligand type in the representable-NR set are randomly split into two folds. Shown are PCCs of the average residue-to-ligand distances across each domain position between the two folds, averaged across 10 repetitions. Relative domain-to-ligand distances across domain positions tend to be highly consistent between structural instances of the same domain–ligand pair. Total number of domain–ligand interactions included in each boxplot are listed above the corresponding distributions.

We next show, in a systematic analysis, that structural interfaces between domains and their ligands in the representable-NR set tend to be conserved. Briefly, we compare the residue-to-ligand distances across different structural instances of a domain–ligand interaction type to each other (see ‘Materials and Methods’ section). We find that analogous positions across domain instances indeed tend to have similar distances to ligands: the median PCC of domain–ligand interactions is 0.98 and the PCC ≥ 0.8 for 91% of domain–ligand interactions (Figure 3 and Supplementary Figure S2). Thus, domains tend to have highly consistent structural interaction interfaces with the same ligand type across instances.

**Figure 4.**
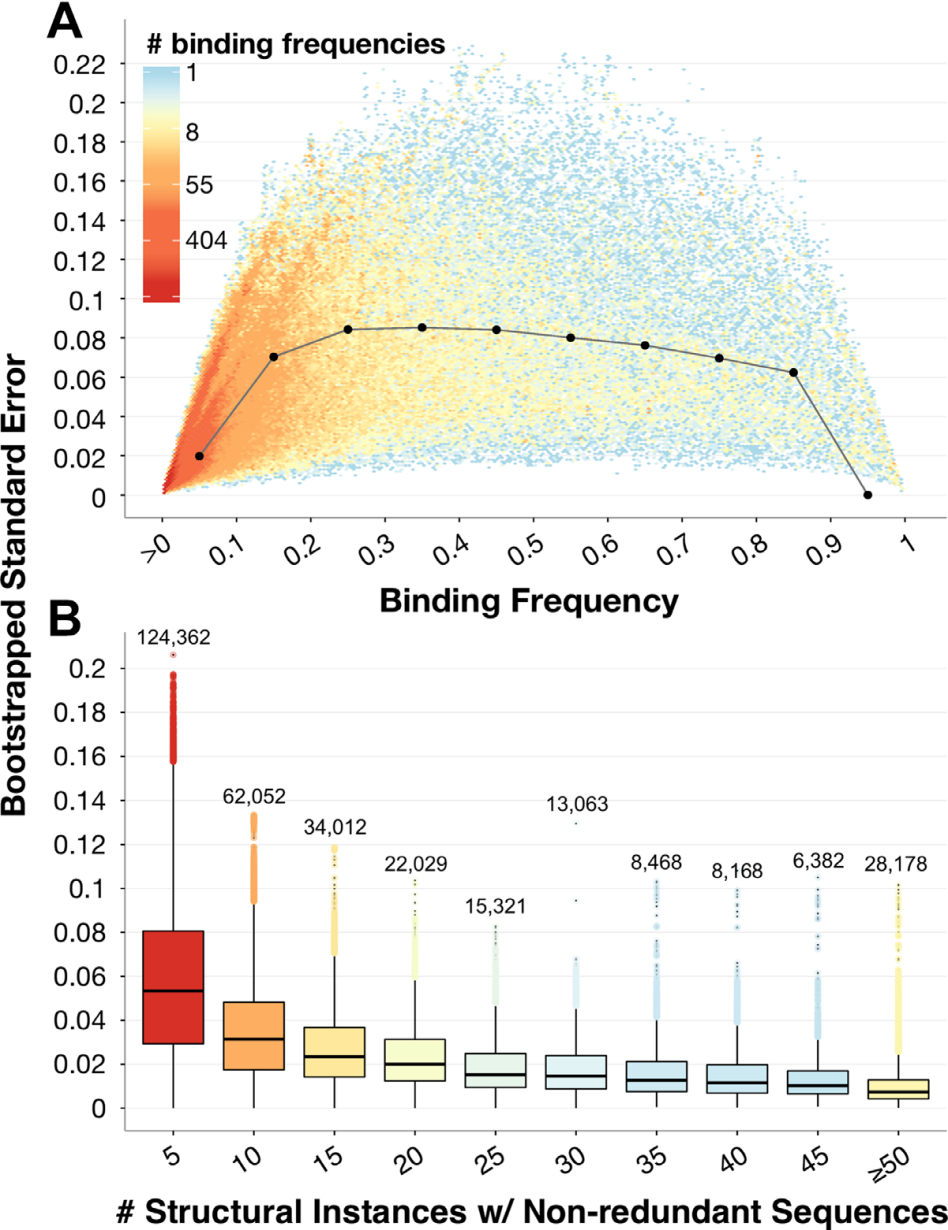
**Bootstrapped standard errors of binding frequencies**. (**A**) For each domain position with a positive binding frequency in each domain–ligand interaction pair in the representable-NR set, we plot its ligand-binding frequency (*x*-axis) and the standard error of this value (*y*-axis), computed as the standard deviation of its ligand-binding frequency as measured over 1000 bootstrap samples. Distribution medians at each binding frequency decile are shown as black dots and are connected by gray lines for visual effect. (**B**) Bootstrapped standard errors decrease as the number of structural domain instances with non-redundant sequences increase, illustrating the ability of our structural aggregation approach to determine how domain positions are generally involved in ligand binding. Boxplots are colored according to the relative size of each distribution; the number of total domain positions, across domain–ligand type pairs, is listed above each boxplot.

As we have just shown, domains generally tend to interact with their ligands in a relatively consistent fashion. However, some interaction domains are also known to have multiple binding modes, where different combinations of domain positions are in contact with the ligand (44,45); these cases cannot be detected by examining structural domain instances in isolation, but may be revealed using our aggregation approach. To get a better idea of how much domain positions vary with respect to their roles in ligand binding, for each domain–ligand type pair in the representable-NR set, we use empirical bootstrapping of structural instances with 1000 repetitions to obtain standard errors of all binding frequencies. Smaller standard errors indicate a domain position’s consistent role in binding, whereas larger bootstrapped standard errors indicate that the measured binding frequency of that position is highly dependent upon the specific groups of structures being considered, and thus the position plays a more variable role in ligand binding. Importantly, standard errors tend to be low for the full range of ligand-binding frequencies (Figure 4A and Supplementary Figure S3a). Moreover, as expected, positions with more extreme binding frequency values (i.e. ≥0.95 or ≤0.05) tend to have lower standard errors. Conversely, positions with intermediate binding frequencies also exhibit more variation in their estimates in bootstrapped samples. We also show that with more sequentially-distinct structural examples of domain–ligand complexes, the standard errors of computed binding frequencies decrease overall (Figure 4B and Supplementary Figure S3b). This latter finding indicates that aggregating information across structural domain instances allows us to infer which positions within domains bind particular ligands more confidently than we could if we were limited to only one or a few structural examples. We note that whereas positions with positive binding frequencies are, by definition, known to interact with ligands in some contexts, positions with 0-value binding frequencies may also sometimes be involved in binding, but are not yet represented in the set of solved structures. Thus, overall, we expect that our aggregation approach will continue to improve as more structures of previously unobserved domain binding modes are experimentally determined.

### Cross-validation highlights power of binding frequencies

**Figure 5.**
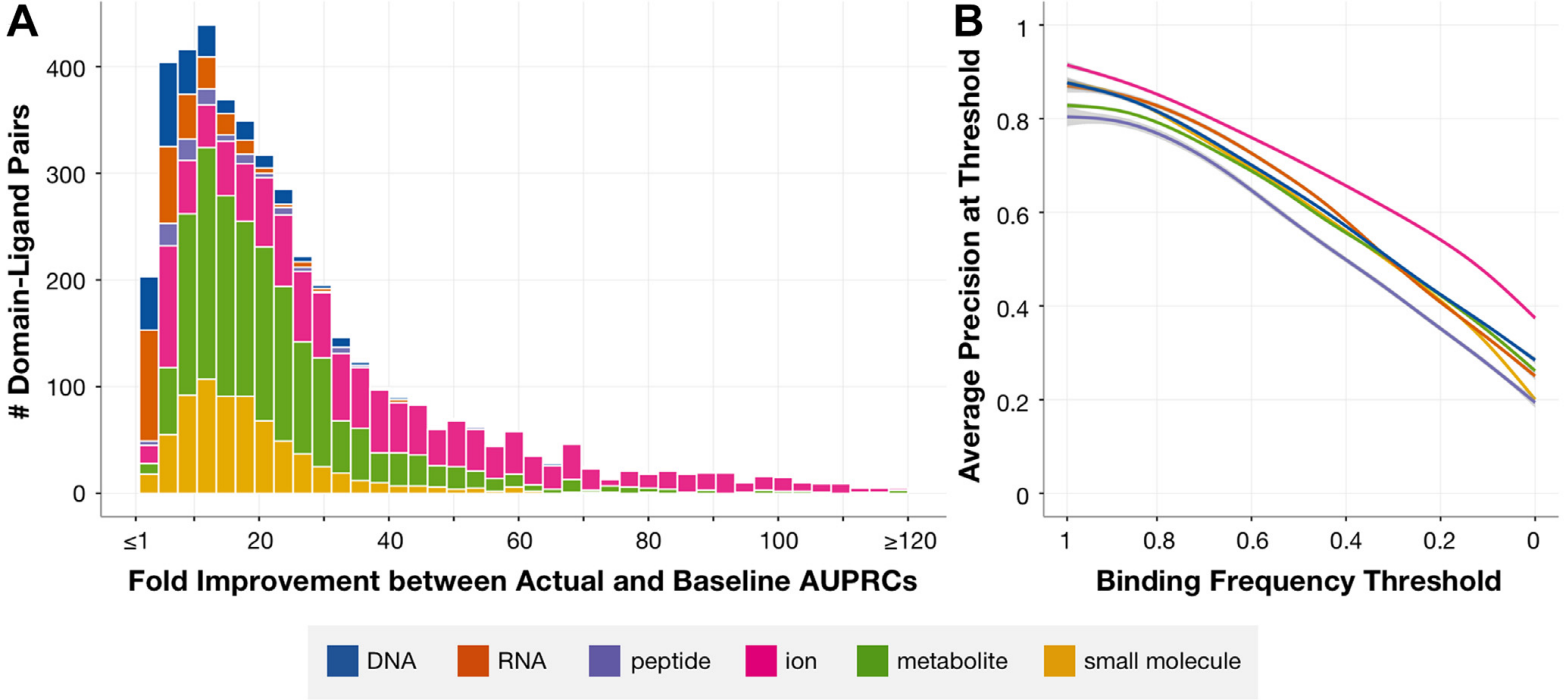
**Cross-validation testing of binding frequencies**. Accuracy of each domain–ligand interaction from the representable-NR set is measured as the average AUPRC in cross-validation with up to 10 folds. (**A**) For each domain–ligand pair, we compute the fold change between the actual AUPRC and a baseline AUPRC corresponding to the fraction of binding positions in the hold-out set. (**B**) We use each positional binding frequency computed across domain–ligand pairs as a threshold to distinguish predicted binding from non-binding domain positions, and we measure the precision achieved in each held-out set of domain–ligand structural instances using each of these thresholds. Shown is the average precision computed across domain–ligand interactions in the representable-NR set at binding frequency thresholds varying from 1 (highest) to 0 (lowest).

We next evaluate how well the binding frequencies for a given domain–ligand type pair indicate positions involved in binding across previously unobserved structural instances. To measure this, we employ cross-validation to compute PR curves for each domain–ligand pair in the representable-NR set, and compare the AUPRCs to corresponding baseline AUPRCs (see ‘Materials and Methods’ section). We find that the average (across folds) actual AUPRCs are typically substantially higher than their corresponding baseline AUPRCs, particularly for domain–ion interactions which tend to involve far fewer domain binding positions and thus have lower baseline AUPRCs (median fold improvement of actual over baseline AUPRCs = 19.9, fold improvement ≥ 5 for 94.2% of domain–ligand interactions, Figure 5A). We also note that the cross-validated precisions for domain–ligand type pairs in the representable-NR set tend to be high across a range of binding frequency cutoffs (Figure 5B). Moreover, when we repeat this process with stricter fold divisions, ensuring that structural instances of domain–ligand interactions in separate folds have <90% sequence similarity, we again find that the improvement of actual over baseline AUPRCs remains high (median fold improvement of actual over baseline AUPRCs = 18.8, fold improvement ≥ 5 for 93.9% of domain–ligand interactions, Supplementary Figure S4). This benchmarking demonstrates that our binding frequencies can be used to infer domain binding positions across previously unseen, sequentially diverse structural instances.

For the remainder of our analysis, we consider a confident set of 12010 domain–ligand interactions from the representable-NR set (involving 2152 distinct domains) that achieve a cross-validated precision ≥0.5 at some binding threshold. Note that though many domain–ligand interactions are structurally consistent across domain instances, ∼25% of domain–ligand interactions in the representable-NR set are not included in the confident set due to the diversity with which they bind their ligands across structural instances. For example, the PAZ (Piwi/Argonaute/Zwille) domain binds RNA using a variety of positions across distinct instances and thus achieves a low cross-validated precision in identifying binding sites in held-out structures.

### Analysis of InteracDome-inferred binding sites in human

We next use our InteracDome resource to infer sites in human proteins that may be involved in interactions with DNA, RNA, peptides, ions, metabolites and small molecules.

#### Domain-based approach doubles coverage of human genes with modeled interactions

**Figure 6.**
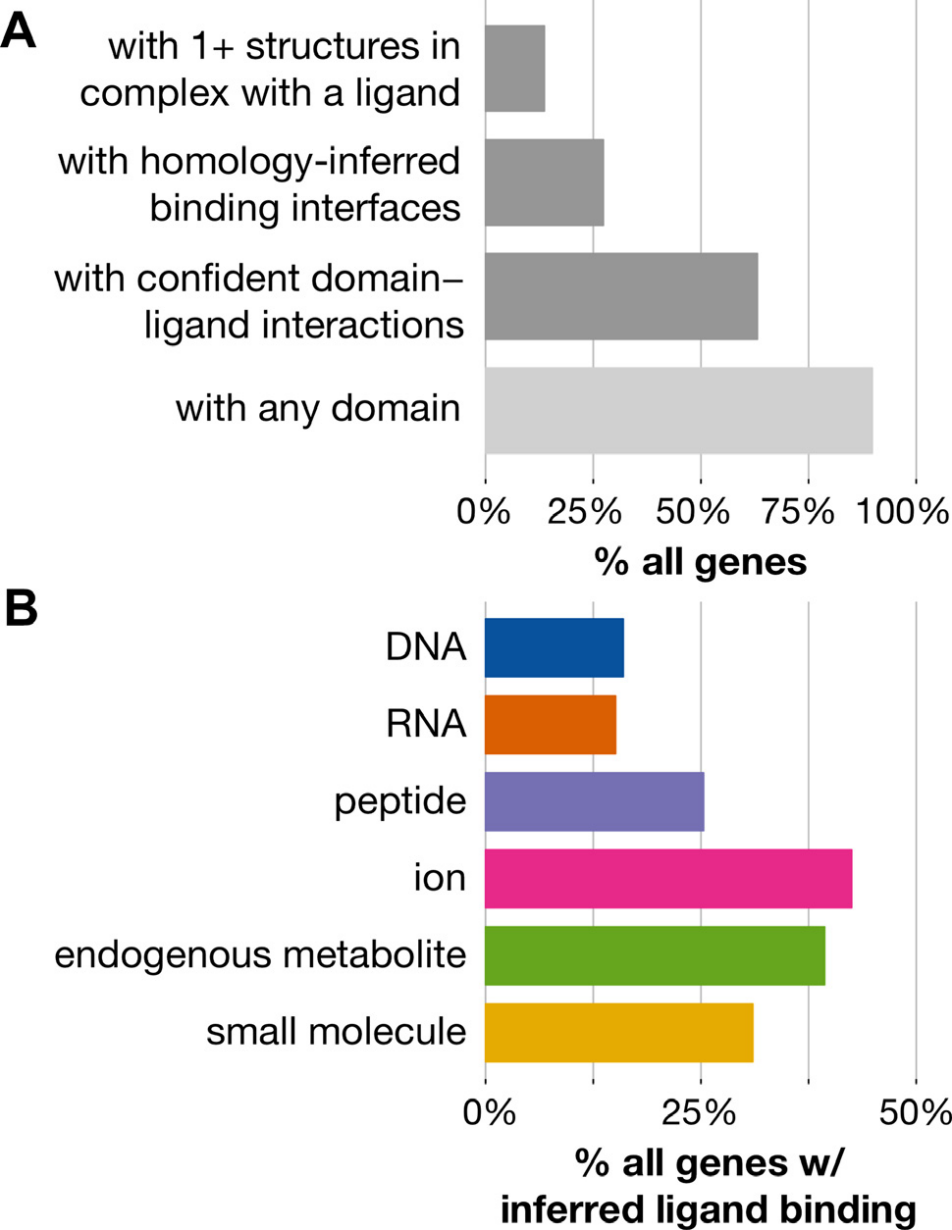
**Interaction domain-based coverage of human genes**. (**A**) Structural and domain-based coverage of 22712 human genes. Dark gray bars indicate ways by which to structurally infer protein interaction interfaces. (**B**) Percentages of genes estimated to interact with specific ligand types using the confident set of domain–ligand interactions.

As of September 2018, only 3099 (13.6% of 22712 total) human genes were associated with biologically relevant protein–ligand complex structures in BioLiP. Homology modeling as described in (13) allows us to infer binding residues in an additional 3110 genes. Together, these two approaches cover 27.3% of all human genes (Figure 6A) (13,46,47). We note that there are an additional 1043 human genes associated with co-complex structures in the Protein Data Bank (PDB) (48), but these proteins are either complexed with non-biologically relevant ligands or with peptides longer than 30 residues (which are not included in our analysis).

Approximately 90% of human genes contain complete instances of ∼6000 Pfam domain families. Of course, not all of these domains have associated structural co-complex information, and thus neither their roles in mediating binding nor which positions within them are involved in ligand binding are known. However, 14335 (63.1%) genes contain InteracDome domain instances with confident interactions or homology-inferred binding interfaces. Including any domain with representable interactions across 5+ or 1+ instances in BioLiP, rather than only domains with confident interactions, respectively covers 64.4 and 69.2% of human genes. Our InteracDome resource thus represents a 2.3- to 2.5-fold increase in coverage over current state-of-the-art approaches to infer putative interaction sites across human genes. Furthermore, our approach covers a diverse range of interaction types, as substantial fractions of these genes contain instances of domains with confident interactions with DNA, RNA, peptides, ions, metabolites and other small molecules (Figure 6B). Altogether, the InteracDome represents a considerable improvement in our ability to infer diverse protein–ligand interaction sites across large numbers of proteins across species.

#### Putative binding sites are depleted of natural variants, enriched for disease mutations

Because the vast majority of proteins’ functions are carried out through specific interactions, even rare DNA variants or mutations that alter interaction-mediating protein residues can have critical impacts in human disease. As such, we expect inferred protein interaction sites to be relatively conserved across healthy human individuals, whereas we would expect these same sites to be perturbed across individuals with disease (13). To determine whether our InteracDome-inferred binding residues exhibit these expected functional constraints, we perform an initial analysis on missense mutations where we consider any protein residue overlapping a domain match state with a corresponding binding frequency that resulted in a cross-validated precision ≥ 0.5 to be a confident ‘putative’ ligand-binding site (see ‘Materials and Methods’ section).

**Figure 7.**
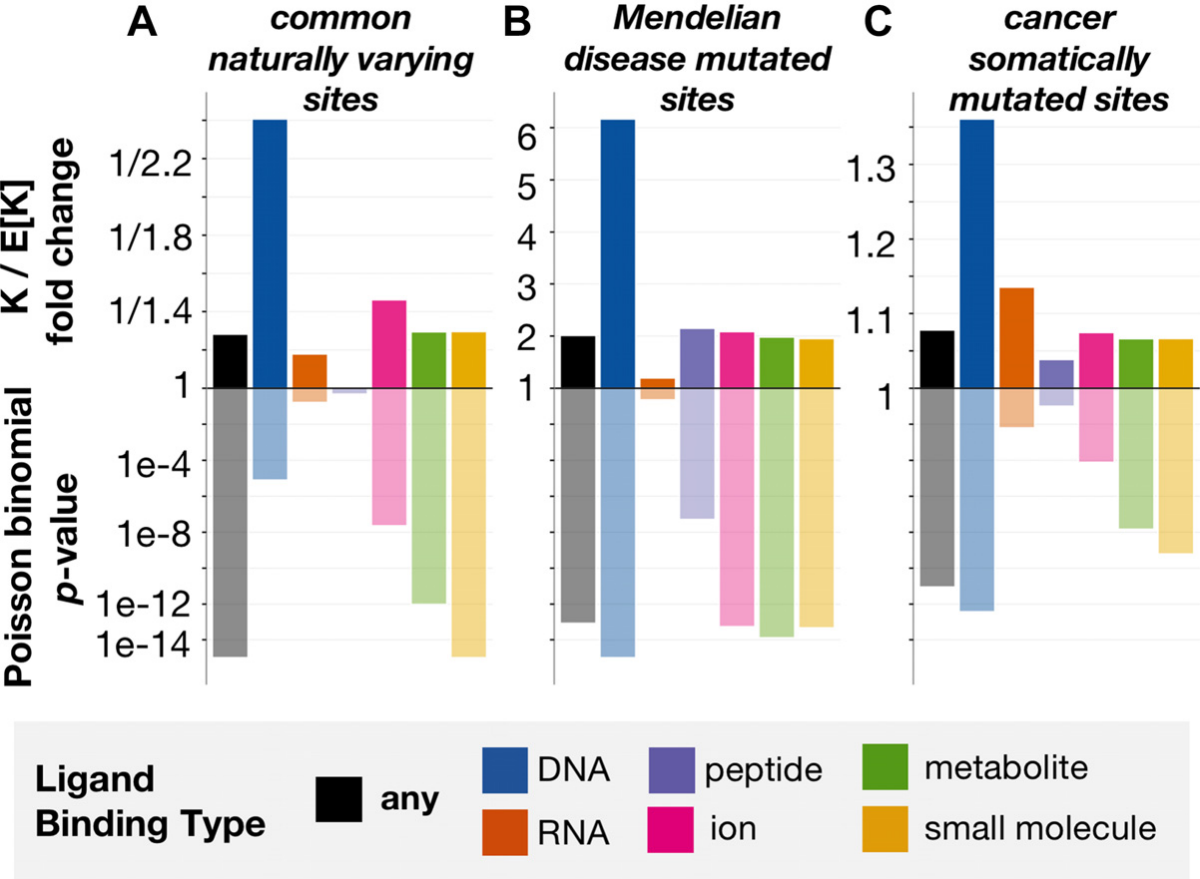
**Natural variants show opposite trends from disease mutations with respect to ligand-binding sites**. Putative ligand-binding sites correspond to protein positions overlapping domain match states whose binding frequencies resulted in a precision of at least 0.5 in cross-validation testing (i.e. confident interactions, see ‘Materials and Methods’ section). Shown on the *y*-axis (top) is the fold change between the observed (K) and expected (E[K]) numbers of InteracDome-inferred putative binding sites (any type, DNA, RNA, peptide, ion, metabolite or small molecule) and other sites of interest (common naturally varying, Mendelian disease mutated, cancer somatically mutated). We compute the significance of this overlap (*y*-axis, bottom) using the Poisson binomial distribution. (**A**) Putative ligand-binding sites exhibit a significant lack of overlap with commonly varying sites across human proteins. (**B**) Conversely, putative ligand-binding sites overlap significantly with sites harboring Mendelian disease mutations. (**C**) Protein sites harboring missense cancer somatic mutations also overlap significantly with putative ligand-binding sites, suggesting that these sites are preferentially altered in human cancers.

We first assess whether commonly varying sites across healthy human individuals tend to globally overlap with putative ligand-binding sites as expected across the human proteome (34). We find that the overlap between commonly varying sites and confident domain-inferred binding sites is significantly *less* than expected by random chance (*P* < 1e-15, Poisson binomial test, Figure 7A). This global trend indicates that sites identified by InteracDome as potentially ligand-binding are generally conserved across healthy individuals, in accordance with what we would expect and further demonstrating the utility of our resource to highlight functionally important protein interaction positions.

We next consider whether protein positions harboring known disease-associated mutations overlap with these same putative binding sites. We uncover that Mendelian disease-mutated sites coincide with putative binding sites far *more* than expected by chance (*P* < 8.4e-14, Poisson binomial test, Figure 7B) (35), in concordance with previous studies of specific diseases (49,50), and that these mutations affect a broad range of ligand-binding sites (Supplementary Table S1).

Finally, we assess whether somatically mutated sites across human cancers overlap with putative binding positions across all human proteins as we might expect by random chance. Others have noted the propensity of cancer mutations to coincide with ligand interaction sites across smaller gene sets and in known driver genes in particular (13,51). We find that over a quarter of the 1209 unique cancer-driving somatic mutations (DoCM, v3.2) (38), for instance, fall into confident putative ligand-binding sites inferred using InteracDome (Supplementary Table S2), even though these sites constitute only ∼2.8% of the entire proteome and ∼14.4% of the proteome covered by a domain with confident interactions (*P* < 8.5e-23, binomial test). Moreover, when we repeat our global, site-based analysis, considering *all* somatically mutated sites across >10000 tumor samples from 33 cancer types and using a much more comprehensive set of inferred binding sites than previous studies, we confirm the same trend. Sites harboring somatic missense mutations tend to coincide with inferred binding sites significantly *more* than expected by random chance (*P* < 9.0e-12, Poisson binomial test, Figure 7C), strongly suggesting that protein interaction perturbation is a frequent mechanism by which somatic mutations contribute to tumor fitness. Indeed, the somatic mutations affecting InteracDome-inferred putative binding sites have higher deleteriousness scores relative to non-binding mutations, as evaluated by various mutational impact predictors (*P* < 1e-14, Fisher’s exact tests, Table 1) (52). However, unlike these other deleteriousness predictors, our InteracDome-inferred binding sites can not only be used to pinpoint potentially disease-relevant mutations, but can also be used to reason about their molecular, mechanistic impacts on protein interaction functionality.

**Table 1.** Fisher’s Exact Tests comparing deleteriousness predictions between binding and non-binding mutations

	# Binding Mutations		# Non-Binding Mutations			
Method	*deleterious*	*tolerated*	*deleterious*	*tolerated*	Odds Ratio	*P*-value
SIFT	23094	9142	486 089	312 266	1.62	∼0
PolyPhen2, HDIV	21208	11801	416 954	400 778	1.73	∼0
PolyPhen2, HVAR	18574	14435	326 362	491 306	1.94	∼0
MutationTaster	6284	3868	129 866	94141	1.18	4e-15
PROVEAN	6411	3429	103 264	114 453	2.07	3e-262
REVEL	3230	6886	47823	175 674	1.72	3e-127
MutPred	6011	3156	92629	109 803	2.26	7e-305

Each distinct somatic mutation in the pan-cancer dataset is classified as either binding (i.e. falls into an InteracDome-inferred, confident putative binding position in at least one human protein) or non-binding. Corresponding deleteriousness scores for each of these mutations were retrieved, where available, from the Database for Nonsynonymous SNPs’ Functional Predictions (v3.5) (52); many mutations analyzed did not have corresponding deleteriousness scores for one or more predictors. Score thresholds to distinguish deleterious from tolerated mutations were set as recommended by each method or to ≥0.5 when not specified for REVEL and MutPred scores.

The respective overlap (and lack thereof) of inferred binding sites with mutated or varying sites is significant even when considering only specific types of ligand binding in turn (Figure 7). Somatically mutated sites in particular appear to overlap with putative DNA-binding sites across proteins significantly more than expected, in accordance with what we know about impaired DNA repair functionality and perturbed regulatory processes in cancer (53). Importantly, we continue to observe the same overall trends for sites exhibiting naturally occurring variation, Mendelian disease mutations, and somatic mutations when we consider alternate precision-based definitions of putative ligand-binding sites from the representable-NR set (Supplementary Figure S5). Overall, given that natural missense variants across healthy populations are depleted in putative binding sites, and Mendelian disease-associated missense mutations as well as somatic missense mutations across cancer tumors are enriched in them, computational approaches that leverage our InteracDome resource to identify perturbed interaction sites are likely to be highly relevant for identifying disease genes and informing disease mechanisms.

## DISCUSSION

We have introduced here a fully automated approach for aggregating protein co-complex structural data in the context of domains to reveal how positions within these domains are generally involved in mediating interactions with DNA, RNA, ions, peptides, metabolites and other small molecules (Figure 1). This collection of 4128 interaction domains, called the InteracDome, can be applied to pinpoint putative interaction sites in various proteins across species; here we show how a subset of 2152 domains with confident ligand interactions can be used to infer functionally relevant interaction sites across the greatest proportion of human genes to date (Figure 6A).

Previously, interaction site information has been transferred from structurally modeled proteins to uncharacterized proteins with similar sequences or subsequences using various homology-based approaches (13,46,47). Sequence motifs have also been semi-manually annotated with highly conserved metal ion binding or catalytic site information for use in identifying functional sites in new proteins, although such approaches are limited due to their rigid sequence match requirements (54). Indeed, the ability of traditional homology-based approaches to infer binding information across larger, more diverse sets of protein sequences using existing structural templates is restricted in general because even as the number of resolved protein structures is increasing, the diversity of their sequences is not. Here, we develop a structurally-aware, domain-based approach to calculate real-valued binding frequencies across individual domain positions. These probabilistically-modeled domain profiles are better able to capture conserved residues required for proper domain folding and thus can be used to accurately transfer binding site knowledge across a far more diverse set of proteins.

Determining the binding positions within these domains represents a challenging task due to biases inherent in structural data: structures often harbor confounding experimental artifacts, are dominated by non-cognate drug interactions, and can have highly redundant sequences to each other (24). We show that by addressing each of these issues in turn, our systematic approach models binding positions across thousands of domains—including nearly all known DNA-, RNA- and peptide-binding domains—that are highly indicative of ligand-binding positions in well characterized domains as well as in structural instances that were held out in cross-validation testing (Figures 2 and 5). Moreover, we find that aggregating domain co-complex structures to develop a general understanding of how a domain participates in interactions is superior to using only one or a few structures for this task (Figure 4B).

Some domains can bind different types of ligands, though one of these may be rare or nonstandard; for instance, the C2H2-ZF and HTH domains predominantly bind DNA but sometimes bind RNA or peptides. Other domains may bind the same ligand type via multiple, diverse binding modes; for example, though the PAZ domain primarily binds RNA, the positions within the domain mediating RNA interactions vary across structural instances. We mitigate these two concerns in our aggregation approach by conservatively restricting domain–ligand interactions to those with substantial structural representation that bind consistently across instances as demonstrated via cross-validation testing (i.e. ‘confident’ domain–ligand interactions).

Substantial previous work has focused on detecting and characterizing the domain–domain interactions that mediate a number of protein–protein interactions across cellular interaction networks (19,55–58). We make the distinction that in our work, we focus not on detecting the particular domain-mediated interfaces between specific protein partners, but rather on understanding which positions within protein domains mediate a variety of interactions in general with nucleic acids, peptides, metabolites, and a wide range of small molecules. Though we do not consider proteins in complex with other whole proteins here, our framework can be naturally extended to characterize domain–domain interactions in more depth.

Overall, we believe that our structural aggregation framework and resultant InteracDome resource lay the groundwork for many future domain-centric analyses and thus will be of broad use for the community. Not only can knowledge of domain binding positions be used to further group, subtype, subdivide or functionally annotate domain families themselves, but the putative interaction sites inferred across proteins using the InteracDome should be relevant in understanding disease etiology. Indeed, we find that these sites are globally conserved across healthy human individuals yet preferentially perturbed in tumor samples and disease populations. As such, we anticipate that future approaches that utilize InteracDome to detect interaction-altering protein coding variants will be a great aid in both prioritizing disease-associated mutations as well as reasoning about their molecular effects.

## Supplementary Material

Supplementary Data

## Notes

**Disclaimer:** The funders had no role in study design, data collection and analysis, decision to publish, or preparation of the manuscript.
