# Supplementary material for: Systematic domain-based aggregation of protein structures highlights DNA-, RNA- and other ligand-binding positions
Source: Nucleic Acids Res. 2018 Dec 7;47(2):582–93. doi: 10.1093/nar/gky1224 (PMC6344845; doi:10.1093/nar/gky1224)
Supplement: Supplementary Data [file gky1224_supplemental_files.pdf]

Supporting Information for

Systematic domain-based aggregation of protein structures highlights DNA-, RNA-, and other ligand-binding positions

Shilpa Nadimpalli Kobren[1] and Mona Singh[2,3,*]

1 Department of Bioinformatics, Harvard Medical School, Boston, MA, USA
2 Department of Computer Science, Princeton University, Princeton, NJ, USA
3 Lewis-Sigler Institute for Integrative Genomics, Princeton University, Princeton, NJ, USA

*To whom correspondence should be addressed. Tel: +1 609-258-2087; Email: mona@cs.princeton.edu

- **Figure S1.** Examples of ligand-proximity scores across domains.
- **Figure S2.** Domain-to-ligand distance consistencies between structural instances with <90% sequence identity.
- **Figure S3.** Standard errors of binding frequencies obtained by bootstrapping groups of structural instances with ≥90% sequence identity.
- **Figure S4.** Cross-validation testing of binding frequencies where structural instances in distinct folds have <90% sequence identity to each other.
- **Figure S5.** Natural variants show opposite trends from disease mutations to overlap with putative ligand-binding sites.
- **Table S1.** Counts of Mendelian disease mutations affecting particular types of ligand-binding sites.
- **Table S2.** Counts of known cancer-driving mutations affecting particular types of ligand-binding sites.
- **References**

Figure S1. Examples of ligand-proximity scores across domains.

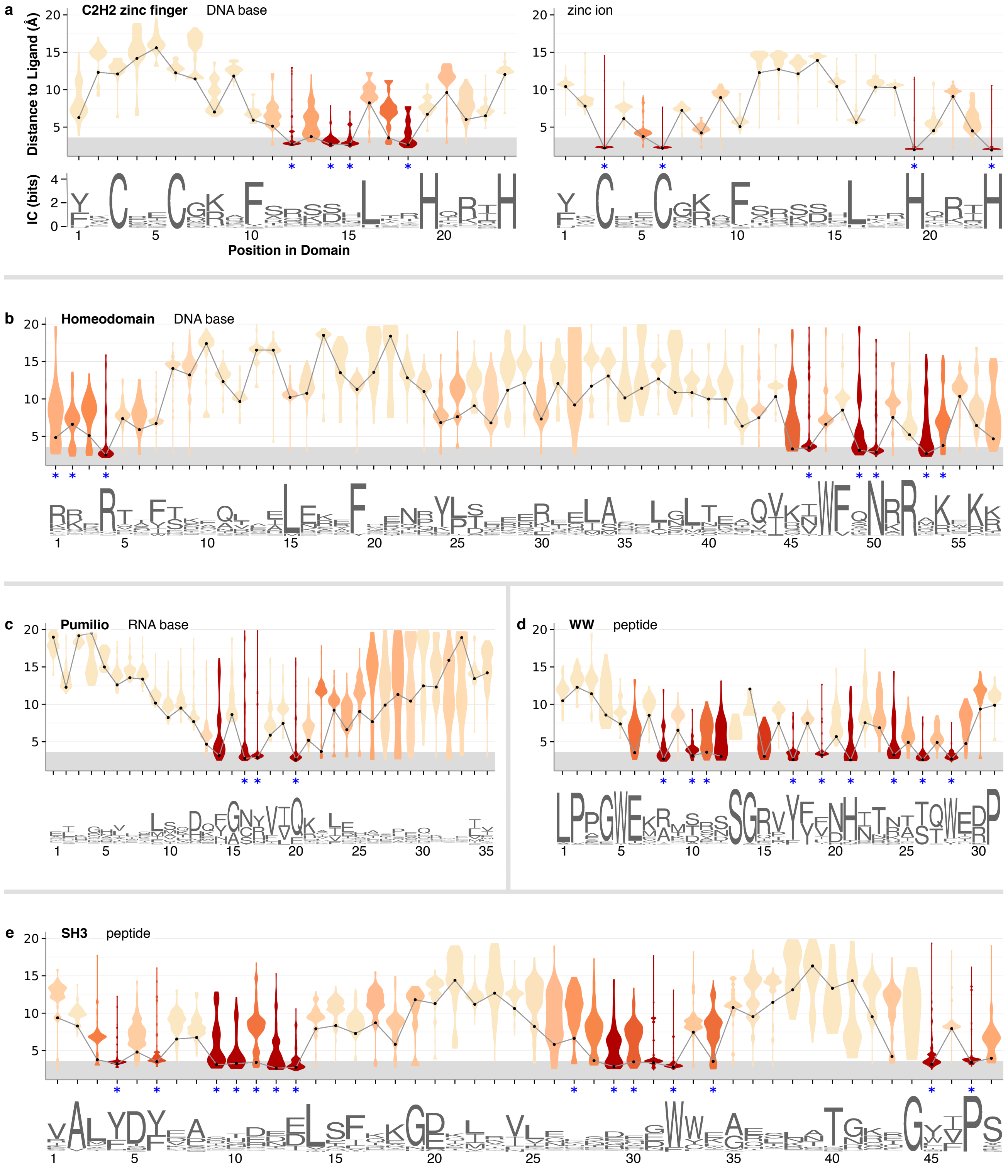

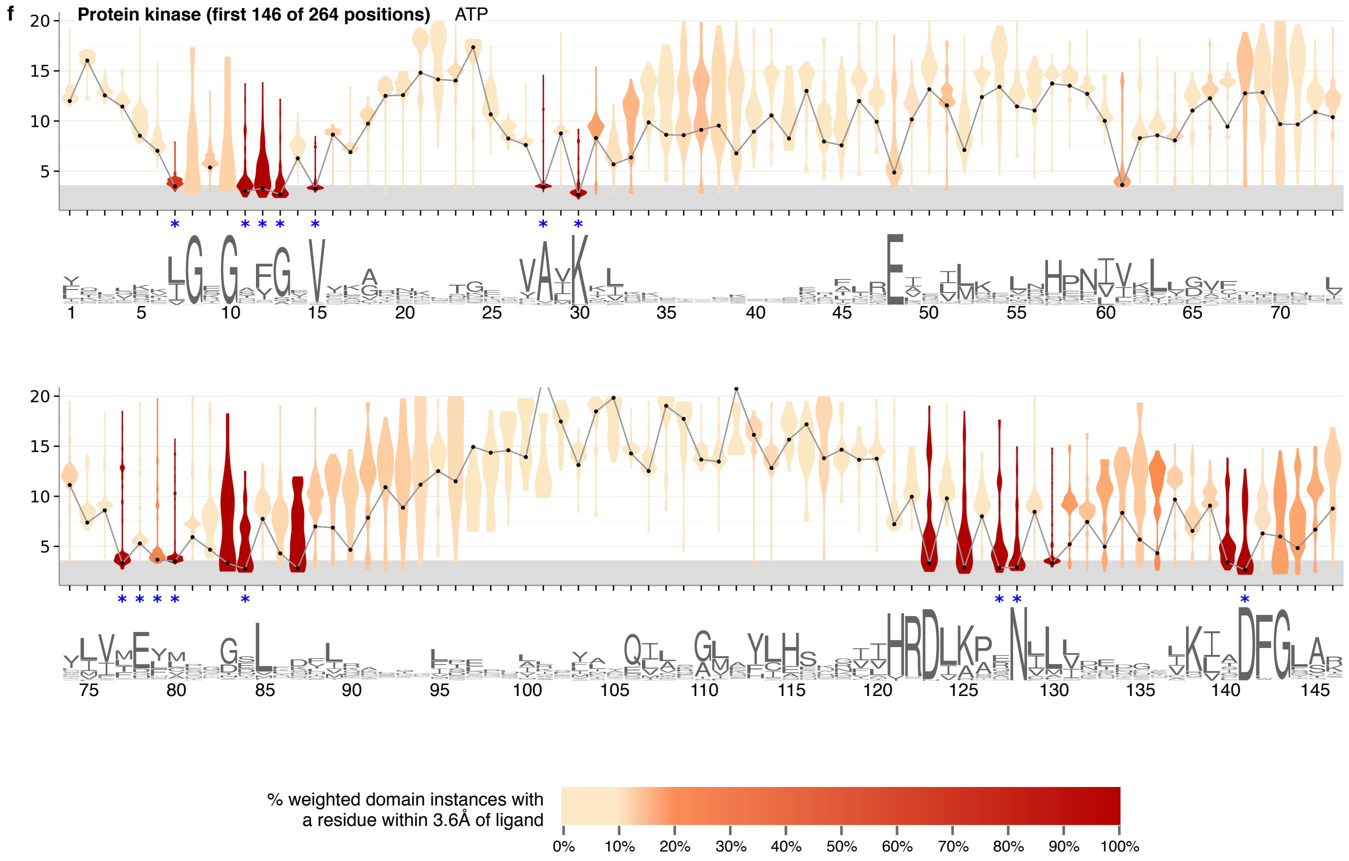

For each position in each interaction domain, we show violin plots depicting the distribution of minimum receptor–ligand distances across instances in BioLiP, colored according to the fraction of the weighted distribution within 3.6Å. Gray lines connect the first deciles of each distribution. The x-axis is labeled with a sequence logo generated from the multiple sequence alignment of domain instances in BioLiP, where column height corresponds to information content. Previously identified ligand-contacting residues [1, 2, 3, 4, 5, 6] are marked with blue asterisks. The x-axis is labeled with a logo generated using Weblogo3 from the multiple sequence alignment of Pfam domain hits across BioLiP. The height of each column in the logos corresponds to the information content (IC) of that column; the logos in (a-f) are scaled equally according to the scale in (a). The particular interaction domains are: **(a)** Cys$_2$-His$_2$ zinc finger domain (PF00096), **(b)** Homeodomain (PF00046), **(c)** Pumilio domain (PF00806), **(d)** WW domain (PF00397), **(e)** SH3 domain (PF00018), and **(f)** the first 146 of 264 positions of protein kinase domain (PF00069).

Figure S2. Domain-to-ligand distance consistencies between structural instances with <90% sequence identity.

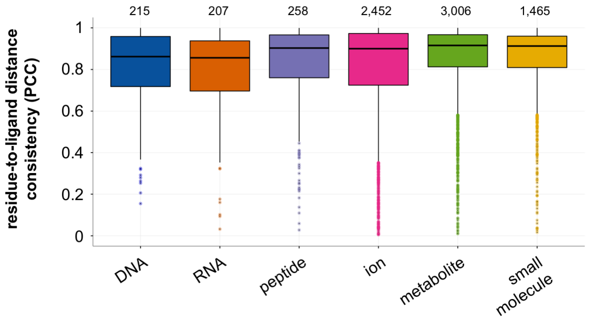

Structural instances across BioLiP for each domain–ligand type are grouped by sequence similarity (≥90% identity), and then these groups are randomly split into two folds. Shown are Pearson's correlation coefficients (PCCs) of the average residue-to-ligand distances across each domain position between the two folds. Total number of domain–ligand interactions included in each boxplot are listed above the corresponding distributions. The median PCC of domain–ligand interactions across these groups is 0.91 and the PCC ≥ 0.8 for 72% of domain–ligand interactions.

Figure S3. Standard errors of binding frequencies obtained by bootstrapping groups of structural instances with ≥90% sequence identity.

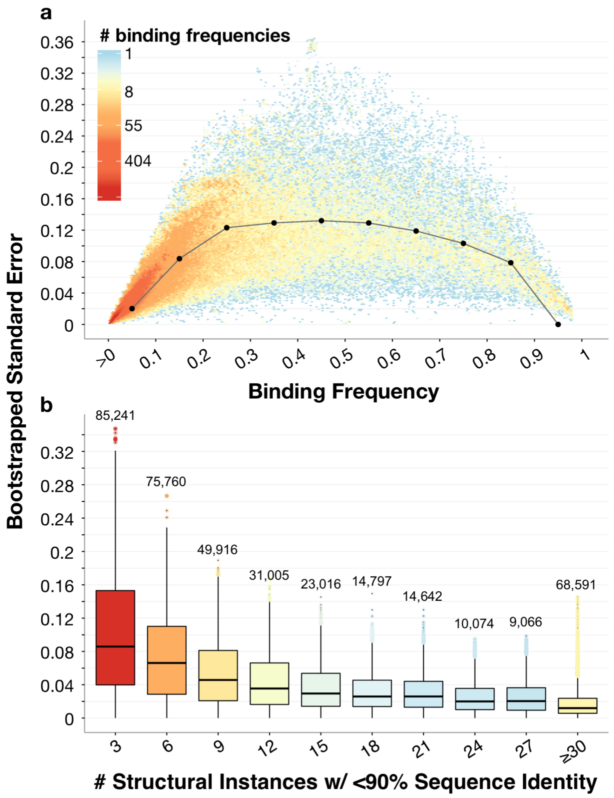

Structural instances across BioLiP for each domain–ligand type are grouped by sequence similarity (≥90% identity), and then these groups are randomly selected with replacement to generate 1,000 empirically bootstrapped sets of structural instances. **(a)** For each domain position with a positive binding frequency in each domain–ligand interaction pair, we plot its ligand-binding frequency (x-axis) and the standard error of this value (y-axis), computed as the standard deviation of its ligand-binding frequency as measured over 1,000 bootstrap samples. Distribution medians at each binding frequency decile are shown as black dots and are connected by gray lines for visual effect. **(b)** Bootstrapped standard errors decrease as the number of domain–ligand structural instances with <90% sequence identity increase. Boxplots are colored according to the relative size of each distribution; the number of total domain positions, across domain–ligand type pairs, is listed above each boxplot.

Figure S4. Cross-validation testing of binding frequencies where structural instances in distinct folds have <90% sequence identity to each other.

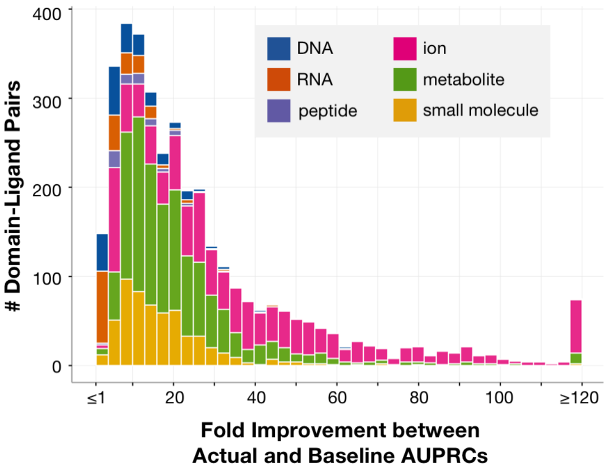

Structural instances across BioLiP for each domain–ligand type are grouped by sequence similarity (≥90% identity), and then these groups are randomly split into up to 10 folds. Accuracy of each domain–ligand interaction with 2+ groups of structural instances is measured as the average area under the precision-recall curve (AUPRC) in cross-validation. For each domain–ligand pair, we compute the fold change between the actual AUPRC and a baseline AUPRC corresponding to the fraction of binding positions in held-out sets.

Figure S5. Natural variants show opposite trends from disease mutations to overlap with putative ligand-binding sites.

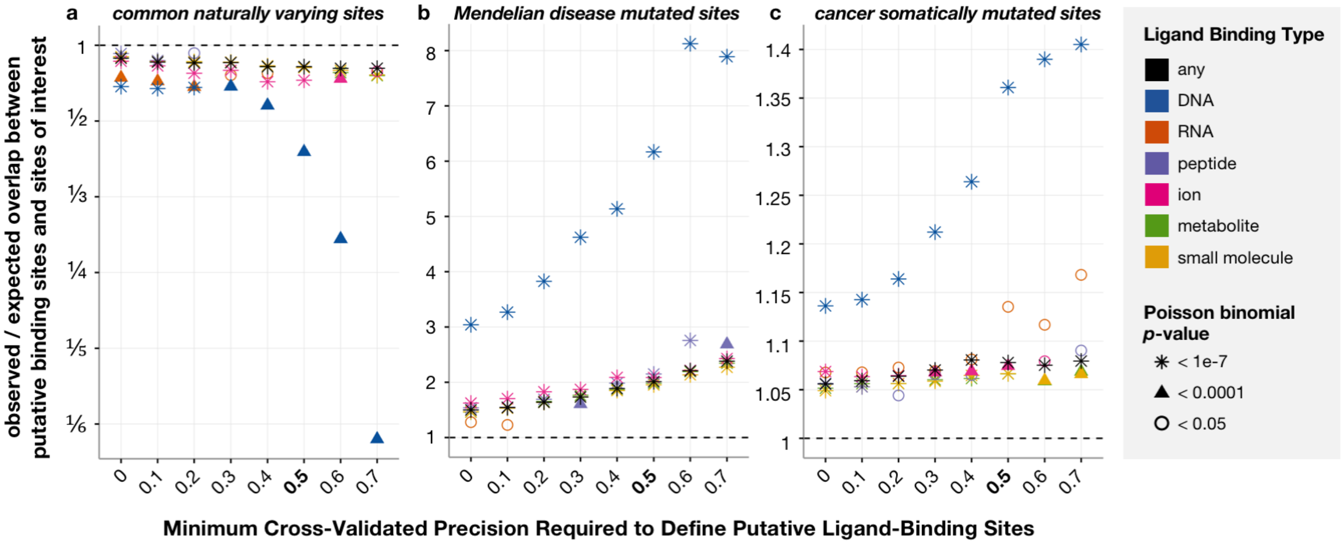

Putative ligand binding sites are inferred across human proteins using domains from the representable-NR set. Specifically, for precision thresholds between 0 and 0.7 (x-axis), protein positions overlapping domain match states whose binding frequencies resulted in at least that precision in cross-validation testing (see Materials and Methods) are considered to be putative binding sites. We compute the significance of the overlap between InteracDome-inferred binding sites and other sites of interest using the Poisson binomial distribution. Bolded along the x-axis is the value used to define putative ligand-binding sites in the main text (i.e., confident interactions); shown along the y-axis is the fold change between the observed number of overlapping sites (K) and the expected number of overlapping sites (E[K]). **(a)** Putative ligand-binding sites exhibit a significant lack of overlap with commonly varying sites across human proteins. Each point corresponds to the fold change between these values for a particular type of ligand-binding site (indicated by its color) for a particular precision-based definition of putative binding site. The shape of each point corresponds to its computed p-value. Fold change values that have corresponding p-values ≥ 0.05 are not shown. **(b)** Conversely, putative ligand-binding sites across human proteins overlap significantly with sites harboring Mendelian disease mutations; points are colored and shaped as in (a). **(c)** Protein sites harboring a missense cancer somatic mutation also overlap significantly with putative ligand-binding sites.

Table S1. Counts of Mendelian disease mutations affecting particular types of ligand-binding sites.

Binding Site Type	representable-NR	confident interactions		
	Total Mutations	*Total Mutations*	*Affected Sites*	*Affected Proteins*
any	4,012	1,276	1,070	441
DNA	314	167	137	60
DNA base	164	82	68	40
DNA backbone	289	119	96	45
RNA	93	21	19	8
RNA base	48	8	8	5
RNA backbone	74	19	18	10
peptide	783	64	57	40
ion	1,040	264	219	121
metabolite	2,720	756	635	285
small molecule	3,142	847	709	305

We consider 30,154 distinct Mendelian-associated missense mutations across 26,434 protein sites in 2,749 proteins. We define putative ligand-binding sites in two ways. First, for each domain–ligand type pair in the representable-NR set, we find matches to the domain in canonical human protein isoform sequences, and consider any protein residue that overlaps with a domain match state whose binding frequency is positive to be a putative ligand-binding site (i.e., representable-NR interactions); 13% of mutations affect these sites. Second, we consider any protein residue that overlaps with a domain match state whose binding frequency resulted in a precision of at least 0.5 in cross-validation testing to be a putative ligand-binding site (i.e., confident interactions, as in the main text); 4% of mutations affect these sites. Columns (from left to right) are the type of ligand interaction, total mutations to affect representable-NR binding sites as described here, total mutations to affect confident binding sites, total number of mutated confident binding sites, and total number of proteins with mutated confident binding sites. Note that the sets of putative nucleic acid base and backbone binding sites are overlapping.

Table S2. Counts of known cancer-driving mutations affecting particular types of ligand-binding sites.

Binding Site Type	representable-NR	confident interactions		
	Total Mutations	*Total Mutations*	*Affected Sites*	*Affected Proteins*
any	484	304	104	31
DNA	73	26	6	1
DNA base	26	4	1	1
DNA backbone	61	22	5	1
RNA	2	1	1	1
RNA base	2	1	1	1
RNA backbone	1	0	0	0
peptide	228	0	0	0
ion	338	60	14	5
metabolite	337	31	11	9
small molecule	363	47	14	8

We consider 1,209 distinct cancer driver missense mutations across 571 protein sites in 128 proteins from the Database of Curated Mutations. We define putative ligand-binding sites in two ways. First, for each domain–ligand type pair in the representable-NR set, we find matches to the domain in the longest human protein isoform sequences, and consider any protein residue that overlaps with a domain match state whose binding frequency is positive to be a putative ligand-binding site (i.e., representable-NR interactions); 40% of mutations affect these sites. Second, we consider any protein residue that overlaps with a domain match state whose binding frequency resulted in a precision of at least 0.5 in cross-validation testing to be a putative ligand-binding site (i.e., confident interactions, as in the main text); 25% of mutations affect these sites. Columns (from left to right) are the type of ligand interaction, total mutations to affect representable-NR binding sites as described here, total mutations to affect confident binding sites, total number of mutated confident binding sites, and total number of proteins with mutated confident binding sites. Note that the sets of putative nucleic acid base and backbone binding sites are overlapping.

References

[1] Wolfe,S.A., Nekludova,L., and Pabo,C.O. (2000) DNA recognition by Cys2His2 zinc finger proteins. *Ann Rev Bioph Biom,* **29**, 183–212.

[2] Noyes,M.B., Christensen,R.G., Wakabayashi,A., Stormo,G.D., Brodsky,M.H., and Wolfe,S.A. (2008) Analysis of homeodomain specificities allows the family-wide prediction of preferred recognition sites. *Cell,* **133**, 1277–1289.

[3] Wang,X., McLachlan,J., Zamore,P.D., and Hall,T.M.T. (2002) Modular recognition of RNA by a human pumilio-homology domain. *Cell,* **110**, 501–512.

[4] Kato,Y., Ito,M., Kawai,K., Nagata,K., and Tanokura,M. (2002) Determinants of ligand specificity in groups I and IV WW domains as studied by surface plasmon resonance and model building. *J Biol Chem,* **277**, 10173–10177.

[5] Saksela,K. and Permi,P. (2012) SH3 domain ligand binding: What's the consensus and where's the specificity?. *FEBS Lett,* **586**, 2609–2614.

[6] Hanks,S.K. and Hunter,T. (1995) Protein kinases 6. The eukaryotic protein kinase superfamily: kinase (catalytic) domain structure and classification. *The FASEB Journal,* **9**, 576–596.
